# Interleukin-1 beta transactivates epidermal growth factor receptor via the CXCL1-CXCR2 axis in oral cancer

**DOI:** 10.18632/oncotarget.5640

**Published:** 2015-10-09

**Authors:** Chia-Huei Lee, Shih-Han Syu, Ko-Jiunn Liu, Pei-Yi Chu, Wen-Chan Yang, Pinpin Lin, Wan-Yu Shieh

**Affiliations:** ^1^ National Institute of Cancer Research, National Health Research Institutes, Zhunan, Taiwan; ^2^ School of Medicine, College of Medicine, Fu Jen Catholic University, New Taipei City, Taiwan; ^3^ Department of Pathology, Show Chwan Memorial Hospital, Changhua City, Taiwan; ^4^ National Environmental Health Research Center, National Health Research Institutes, Zhunan, Taiwan; ^5^ Division of Environmental Health and Occupational Medicine, National Health Research Institutes, Zhunan, Taiwan

**Keywords:** interleukine-1beta, epidermal growth factor receptor (EGFR), CXCL1, CXCR2, oral squamous cell carcinoma (OSCC)

## Abstract

Hyperactivation of the epidermal growth factor receptor (EGFR) pathways and chronic inflammation are common characteristics of oral squamous cell carcinoma (OSCC). Previously, we reported that OSCC cells secrete interleukin-1 beta (IL-1β), which promotes the proliferation of the oral premalignant cell line, DOK, and stimulates DOK and OSCC cells to produce the chemokine CXCL1. CXCL1 functions through CXCR2, a G protein-coupled receptor that transactivates EGFR in ovarian and lung cancers. We hypothesized that IL-1β transactivates EGFR through the CXCL1–CXCR2 axis in OSCC. In this study, we demonstrated that tyrosine phosphorylation of EGFR is crucial for the IL-1β-mediated proliferation and subsequent bromodeoxyuridine (BrdU) incorporation of DOK cells because the EGFR inhibitors AG1478 and erlotinib inhibit these abilities in a dose-dependent manner. Addition of IL-1β instantly enhanced CXCL1 expression and secretion (within 15 min) in the DOK and OSCC cell lines. Furthermore, tyrosine phosphorylation of EGFR was significantly enhanced in DOK (1 h) and OSCC (20 min) cell lines after IL-1β treatment, and both cell lines were inhibited on the addition of an IL-1 receptor antagonist (IL-1Ra). CXCL1 treatment resulted in EGFR phosphorylation, whereas the knockdown of CXCL1 expression by lentivirus-mediated shRNA or the addition of the CXCR2 antagonist SB225002 dramatically reduced IL-1β-mediated EGFR phosphorylation and proliferation of DOK cells. Neutralizing antibodies against IL-1β or CXCL1 markedly inhibited the constitutive or IL-1β-induced tyrosine phosphorylation of EGFR in OSCC cells. IL-1β transactivates EGFR through the CXCL1-CXCR2 axis, revealing a novel molecular network in OSCC that is associated with autocrine IL-1β and EGFR signaling.

## INTRODUCTION

Among head and neck cancers (HNCs), oral squamous cell carcinoma (OSCC) the sixth most common cancer and is a rapidly growing health problem. Late prognosis, lymph node metastasis, and recurrence lead to a particularly poor 5-year survival rate [1]. Chronic inflammation augments the initiation, promotion, and progression of OSCC [2, 3]. Interleukin-1 beta (IL-1β), a critical mediator of chronic inflammation, has been identified as a salivary biomarker for OSCC detection [4]. Consistent with this, in our previous study, we identified that an increased IL-1β level was associated with the increased severity of oral malignant transformation in a mouse OSCC model [5]. In addition, we showed that OSCC cell lines secrete a substantial amount of IL-1β, which promotes tumor proliferation in an autocrine manner. However, the mechanisms underlying IL-1β-triggered OSCC proliferation have not been fully elucidated.

Epidermal growth factor receptor (EGFR, also known as ErbB1 or HER1) overexpression or activation is another condition associated with OSCC. EGFR overexpression has been reported in more than 80% of OSCCs [6, 7] and occurs at the initial stage of carcinogenesis. EGFR is a transmembrane glycoprotein with a cytoplasmic domain that exhibits tyrosine kinase activity [8]. By regulating downstream pathways, EGFR is vital for proliferation, migration, and angiogenesis [9]. Shin et al. reported that EGFR expression increases as the oral tissue progresses from normal mucosa to hyperplasia to dysplasia, and eventually to SCC [10]. Mak et al. demonstrated that a high EGFR expression was observed in oral premalignant (OPM) lesions, and EGFR gene copy number in OPM cells is higher than that in normal mucosa [11]. In a study cohort of 145 patients with OPM lesions, a high EGFR expression was associated with increased oral cancer risk and reduced time to oral cancer, suggesting a promoting role of EGFR in oral malignant transformation and the prognostic implication of EGFR expression [12]. In addition, activation of EGFR signalling is associated with tumor aggression events, such as metastasis and chemotherapy resistance, and is a poor prognostic biomarker in oral cancer [13, 14]. Accordingly, EGFR is a promising target for treating oral cancer. Cetuximab (erbitux) is a chimeric human-murine anti-EGFR-specific monoclonal antibody and currently approved in combination with radiotherapy or chemotherapy. However, the overall increased response of adding cetuximab to radiotherapy or chemotherapy is generally not higher than 20% [17, 18], and only few patients exhibit a long-term response. The response on using cetuximab as a single agent is lower than 13% response, and only a short-term response (2–3 m) was observed [18]. Understanding the mechanism of intrinsic EGFR activation in OSCC may help to identify new molecular targets for treatment.

Previously, we showed that IL-1β activated an oncogenic cytokine network in DOK cell line and TW2.6 OSCC cell lines by stimulating IL-6, IL-8, and CXCL1 production [5]. CXCL1 is a chemokine acting as an oncogenic factor in several cancers, such as prostate cancer, melanomas, and gliomas [19–21]. CXCL1 selectively binds to CXCR2, a G-protein-coupled receptor (GPCR) that has been shown to transactivate EGFR in ovarian cancer and non-small cell lung cancer [22–24]. We hypothesize that IL-1β can modulate EGFR activation through IL-1β-dependent CXCL1 expression which promotes carcinogenesis. To examine our hypothesis, in this study, we investigated the effect of IL-1β on EGFR activation and CXCL1 production in OSCC.

## RESULTS

### Proliferation in response to IL-1β is EGFR dependent

**Figure 1:**
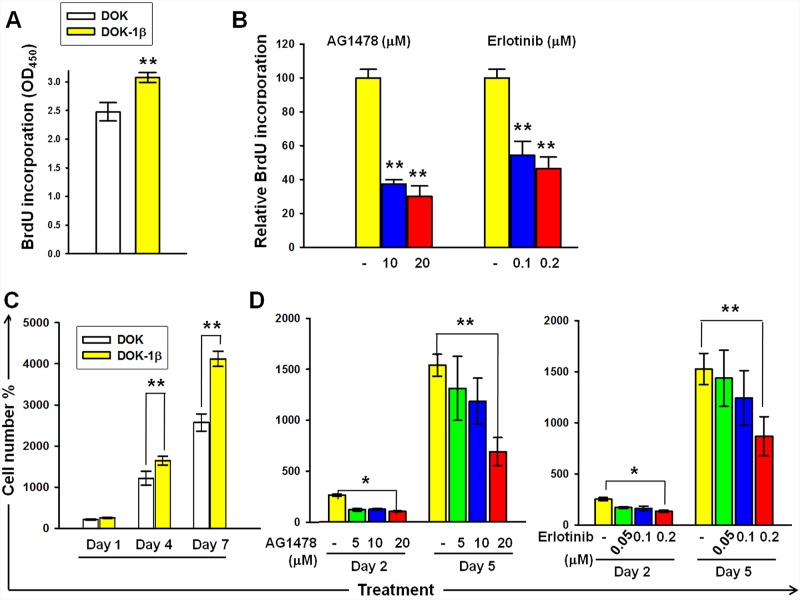
DOK proliferation in response to IL-1β is dependent on EGFR kinase activity **A.** BrdU incorporation was higher in DOK-1β cells than in the DOK cells. **B.** EGFR-TKIs AG1478 and erlotinib suppressed BrdU incorporation in DOK-1β cells in a dose-dependent manner. **C.** Increased DOK-1β cell proliferation was measured using the MTT assay. **D.** Dose-dependent inhibition effects of AG1478 and erlotinib on the DOK-1β proliferation were measured using the MTT assay. Cells were seeded at 5 × 10^4^ (BrdU assay) or 5 × 10^3^ (MTT assay) cells per well on 96-well plates. The next day, cells were treated with DMSO (controls), AG1478, and erlotinib at the indicated concentrations. After a 4-hour treatment, cells were cultured in a normal growth medium, and proliferation assays were performed either after 1 day of treatment, using the BrdU assay, or after 2 and 5 days of treatment, using the MTT assay. Data are expressed as mean ± SD of three independent experiments, where each experiment has been performed in six replicates. *P ≤ 0.01 versus untreated controls.

EGFR plays a major regulatory role in the cell proliferation of human keratinocytes and is associated with OSCC. Previously, we demonstrated that DOK cells treated with IL-1β (1 ng/mL) for 7 days (DOK-1β) exhibit enhanced proliferative properties and Akt phosphorylation [5]. The Akt pathway is one of the EGFR downstream pathways strongly associated with cell proliferation; therefore, we examined whether EGFR activities were involved in IL-1β-mediated DOK proliferation. As depicted in Figure 1A and 1C, increased DOK-1β proliferation was confirmed on performing BrdU and MTT assays, respectively. DOK-1β cells exhibited a dose-dependent reduction on BrdU incorporation when cultured with increasing doses of EGFR tyrosine kinase inhibitor (TKI) AG14778 (10 and 20 μM) and erlotinib (0.1 and 0.2 μM) (Figure 1B). The inhibitory effect of AG14778 and erlotinib on the proliferative properties of DOK-1β cells was confirmed using the MTT assay (Figure 1D). These results suggested that activation of the EGFR signaling pathway is involved in the IL-1β-mediated proliferation of DOK cells.

### IL-1β transactivates EGFR and stimulates CXCL1 expression in DOK and OSCC cell lines

**Figure 2:**
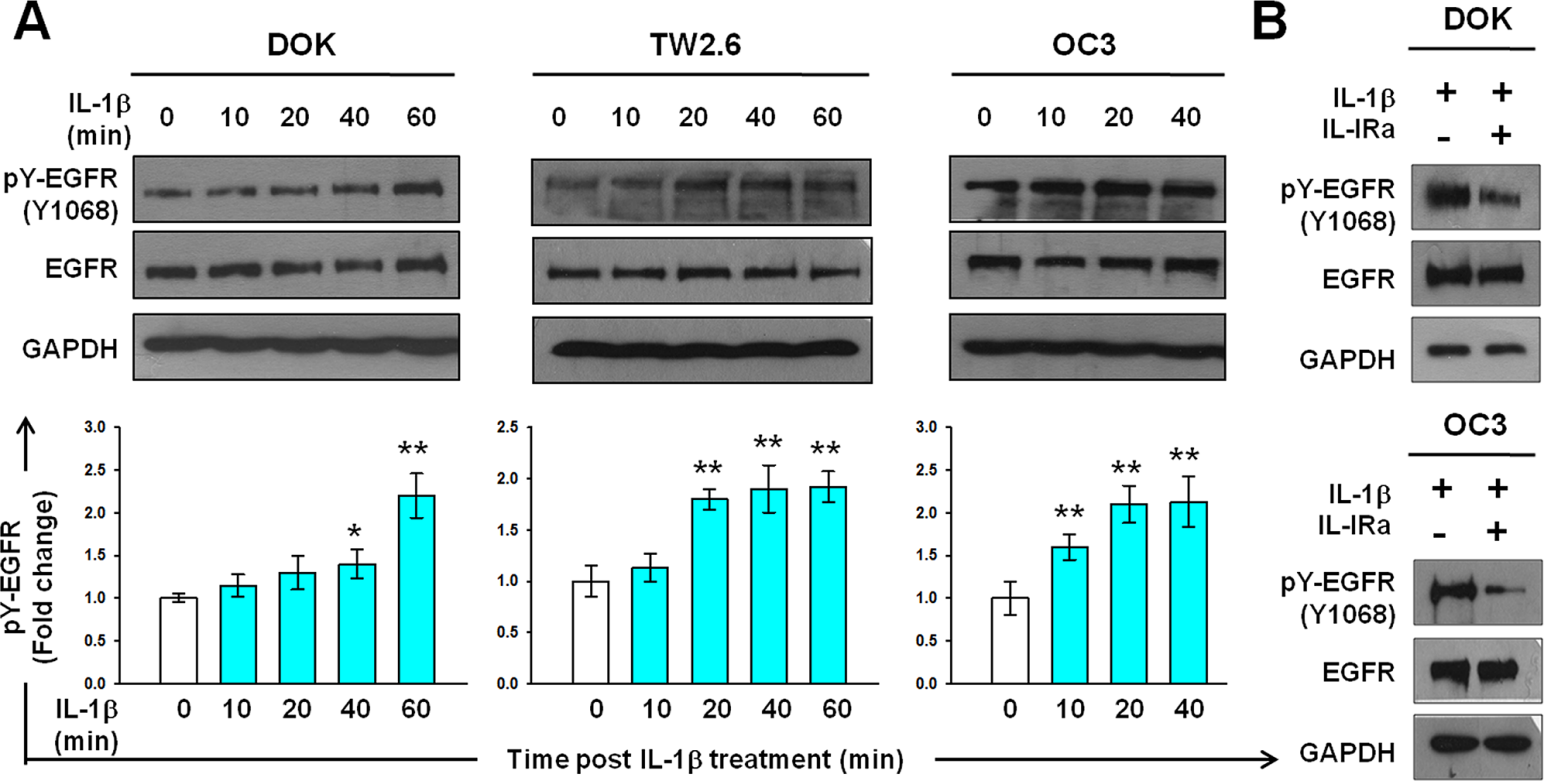
IL-1β induces EGFR tyrosine phosphorylation in DOK and OSCC cell lines **A.** Kinetics of EGFR tyrosine phosphorylation in response to IL-1β in DOK and OSCC cell lines. Cells were cultured in serum-free medium for 24 hours and then treated with IL-1β (1 ng/mL, blue bar). Cell lysates were harvested on ice at the indicated times after IL-1β addition. Equal amounts of whole cell lysates were analyzed for phospho-EGFR (pY-EGFR) and total EGFR protein levels using Western blotting. A representative blot is shown in the top panel. Densitometric analyses were performed to quantify the intensity of the blots. Data are expressed as mean ± SD (*n* = 3). *P < 0.05 versus basal activation. **B.** IL-1 receptor antagonist (IL-1Ra) inhibits IL-1β-mediated EGFR tyrosine phosphorylation. Cells were pretreated with or without 100 μg/mL of IL-Ra for 2 hours prior to IL-1β (1 ng/mL) addition. Cell lysates were harvested on ice 16 hours post IL-1β addition. Whole cell lysates were analyzed using Western blotting, as described in (A).

To examine whether IL-1β induces EGFR activation, we performed western blotting analysis for tyrosine phosphorylation of EGFR in DOK and OSCC cells after IL-1β treatment. As shown in Figure 2A, a marked increase in EGFR tyrosine phosphorylation was observed in DOK cells 1 hour after IL-1β addition. EGFR tyrosine phosphorylation in response to IL-1β increased significantly above basal levels in the TW2.6 and OC3 cells at 20 and 10 minutes, respectively. In both OSCC cell lines, IL-1β-induced EGFR tyrosine phosphorylation further increased at 40 minutes (Figure 2A). Notably, intrinsic EGFR activation was more intense in the OC3 cells, in which IL-1β secretion is high, than in the TW2.6 cells, in which IL-1β secretion is low [5]. To verify the inductive effect of IL-1β in EGFR activation, EGFR tyrosine phosphorylation of DOK and OC3 cells was measured after a 2-h preincubation with 0.5 μg/mL of IL-1Ra followed by IL-1β treatment for 40 minutes. EGFR tyrosine phosphorylation in response to IL-1β was markedly inhibited by the IL-1Ra in DOK and OC3 cells (Figure 2B). This suggests that EGFR tyrosine phosphorylation in response to IL-1β is primarily governed by IL-1 receptor signaling.

**Figure 3:**
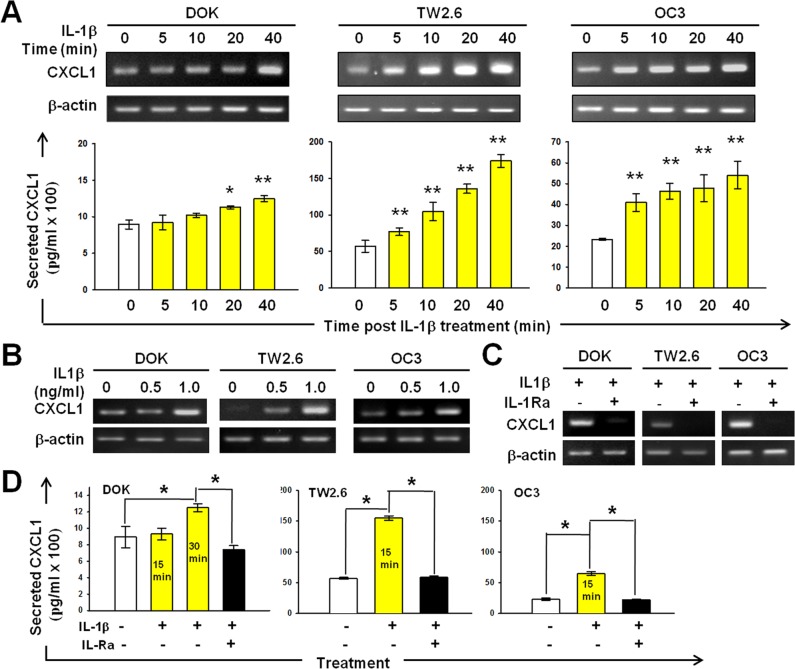
IL-1β-induced CXCL1 production in DOK and OSCC cell lines **A.** Kinetics of CXCL1 mRNA expression in response to IL-1β in DOK and OSCC cell lines. **B.** Dose-dependent induction of CXCL1 mRNA expression by IL-1β in DOK and OSCC cell lines. Cells were cultured in serum-free medium for 24 hours and then treated with IL-1β (1 ng/mL, yellow bar) at the indicated times (A) or for 24 hours with indicated concentrations of IL-1β (B). The total RNA was isolated and CXCL1 and β-actin (internal control for normalization) mRNA levels were determined using real-time RT-PCR analysis. Amplification products of CXCL1 and β-actin are shown in the upper panel. Data are expressed as mean ± SD (*n* = 3). *P < 0.01 versus PBS controls. **C.** IL-1Ra inhibits IL-1β-induced CXCL1 mRNA expression. Cells were cultured in serum-free medium for 24 hours and then pretreated with 100 μg/mL of IL-1Ra for 2 hours prior to IL-1β (1 ng/mL) addition. Cells without IL-1Ra pretreatment were used as controls. The total RNA was isolated 24 hours post IL-1β addition and subjected to real-time RT-PCR analysis. **D.** Effect of IL-1β and IL-1Ra on CXCL1 secretion by DOK and OSCC cells. Cells were cultured in the absence (white bars) or presence of IL-1β with (black bars) or without (yellow bars) IL-1Ra (100 μg/mL) pretreatment for 2 hours. The culture medium was analyzed for secreted CXCL1 protein levels using ELISA 15 min or 30 min after IL-1β addition. Results are expressed as mean ± SD (*n* = 3). *P < 0.05 versus PBS controls.

To investigate whether IL-1β induces EGFR tyrosine phosphorylation through CXCL1-CXCR2 axis, we examined the effect of IL-1β on the CXCL1 expression and secretion. We measured the CXCL1 mRNA expression in DOK, TW2.6, and OC3 cells within 40 min after IL-1β (1 ng/mL) addition. In the DOK cells, upregulated CXCL1 expression was observed 20 and 40 minutes after IL-1β addition (Figure 3A). CXCL1 expression was markedly upregulated in IL-1β-treated TW2.6 and OC3 cells immediately after IL-1β addition (5 min after addition) and a gradual increase was further observed (Figure 3A). As shown in Figure 3B, IL-1β induced CXCL1 expression in a dose-dependent manner and was inhibited by IL-1Ra (2-h preincubation with 0.5 μg/mL of IL-1Ra, Figure 3C). CXCL1 belongs to a group of highly secreted proteins. DOK, TW2.6, and OC3 cell supernatants were analyzed using ELISA for CXCL1 protein secretion after 15 min incubation with 1 ng/mL of IL-1β with or without pretreatment with 0.5 μg/mL of IL-1Ra. As shown in Figure 3D, IL-1β markedly upregulated CXCL1 protein secretion in TW2.6 and OC3 cells (15 min after addition). In the DOK cells, the CXCL1 secretion after IL-1β addition was unchanged at 15 minutes but increased significantly at 30 minutes. These results are consistent with our previous observations, in which the EGFR phosphorylation in the TW2.6 and OC3 cells significantly increased 20 minutes after IL-1β addition; however, no significant increase was observed until 40 and 60 minutes in the DOK cells (Figure 2A). Preincubation with IL-1Ra resulted in a drastically reduced CXCL1 secretion in all cell types. Overall, these data suggest that IL-1β stimulates CXCL1 expression and secretion in DOK and OSCC cells and, in turn, transactivates EGFR through the CXCL1–CXCR2 axis.

### Expression of IL-1β, CXCL1, and CXCR2 in OSCC

**Figure 4:**
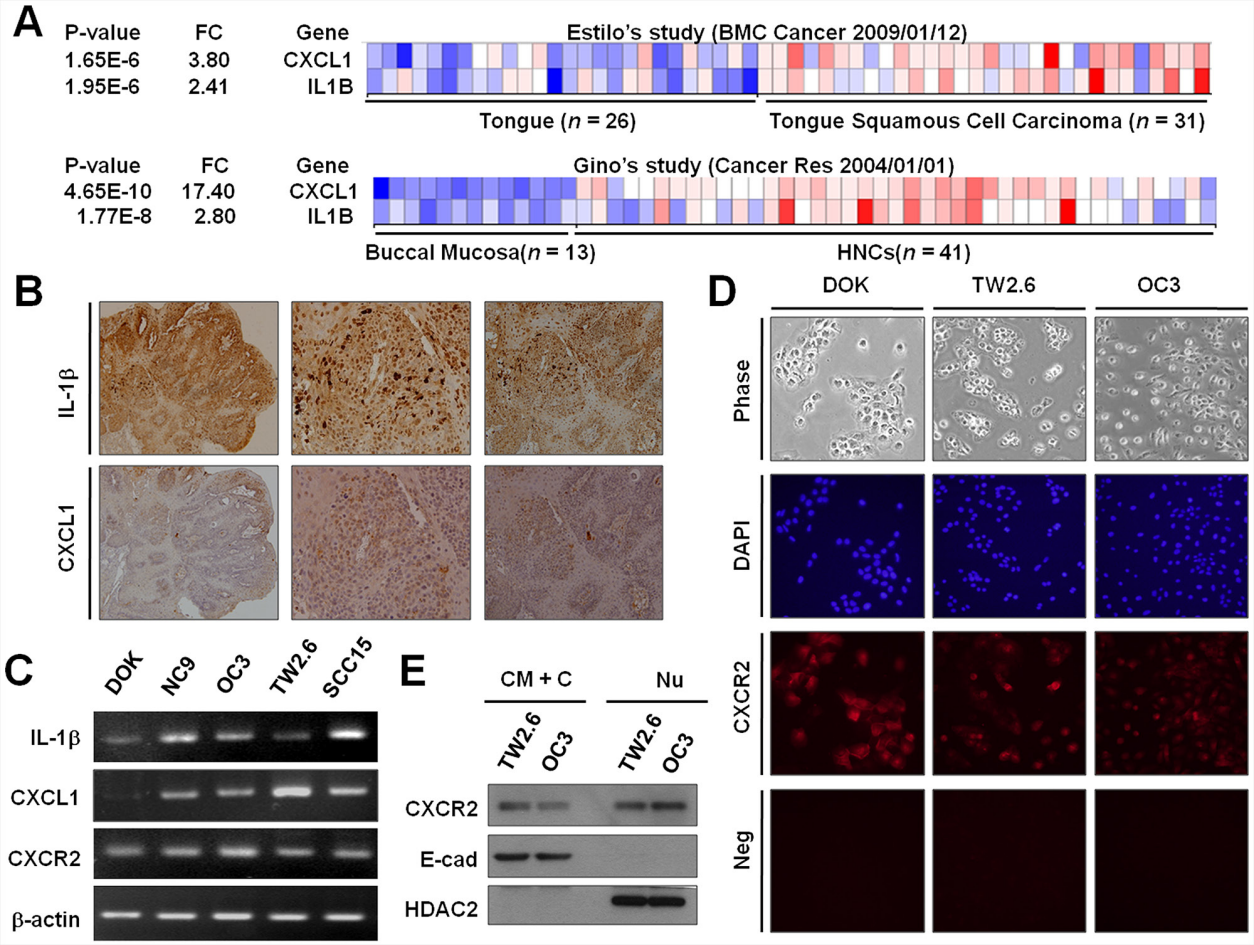
Expression of IL-1β, CXCL1, and CXCR2 in OSCC **A.** Coexpression of IL-1β and CXCL1 in OSCC tumors in human (A) and mouse **B.** (A) The pro-IL-1β and CXCL1 expression profiles of a normal tongue and tongue cancer (upper panel) and normal buccal mucosa and HNCs (lower panel) were obtained from publicly available microarray datasets in the Oncomine database (www.oncomine.com). (B) Immunohistochemistry for IL-1β (upper panel) and CXCL1 (lower panel) was performed on the tongue tissues of a mouse OSCC model. **C.** The mRNA expression of IL-1β, CXCL1, and CXCR2 in DOK and OSCC cells were measured using quantitative RT-PCR. **D.** The immunofluorescent staining of CXCR2 expression in DOK and OSCC cells. Neg, parallel experiments without antibodies specific to CXCR2 as negative controls. **E.** CXCR2 is expressed in the cytoplasmic membrane (CM) and nuclear (Nu) envelope of OSCC cells. Proteins were harvested from the CM and cytoplasmic (C) fraction, and the Nu fractions were subjected to Western blotting for measuring CXCR2 protein expression. E-cad and (HDAC2) were used as molecular markers for CM+C and Nu fractions, respectively.

In our previous study [5], we demonstrated IL-1β upregulation in mouse OSCC. If IL-1β could induce CXCL1 production *in vivo*, CXCL1 and IL-1β may be coexpressed in OSCC biopsies. We thus examined whether CXCL1 was expressed in IL-1β-positive OSCC. We surveyed the publicly available microarray studies in the Oncomine database (www.oncomine.org) and observed that IL-1β and CXCL1 were upregulated in tumor samples compared with normal tongue and normal buccal mucosa samples (*P* < 0.001 for both) (Figure 4A) [25, 26]. Immunohistochemical examination and quantitative real-time PCR (RT-PCR) were conducted. We observed that IL-1β and CXCL1 coexpressed in mouse OSCC samples (Figure 4B) and human OSCC cell lines (Figure 4C). Notably, both IL-1β and CXCL1 were undetectable in normal mouse oral tissues (data not shown). Supporting this finding, DOK cell line expressed lower IL-1β and CXCL1 levels than the other OSCC cell lines tested (Figure 4C). To determine CXCR2 expression in OSCC cells, quantitative RT-PCR, immunofluorescent staining, and western blot analysis and were performed. Our data indicated that CXCR2 mRNA was expressed in all the cell lines examined (Figure 4C), and CXCR2 proteins were detected in DOK, TW2.6, and OC3 cells (Figure 4D). Results from western blotting identified that CXCR2 presents in cytoplasmic membrane of TW2.6 and OC3 cells (Figure 4E). Overall, these results not only support IL-1β-induced CXCL1 expression but also suggest that CXCL1 could exert its activity of EGFR transactivation by binding to CXCR2 in DOK and OSCC cells.

### CXCL1 induces EGFR tyrosine phosphorylation and contributes to IL-1β-mediated DOK proliferation

**Figure 5:**
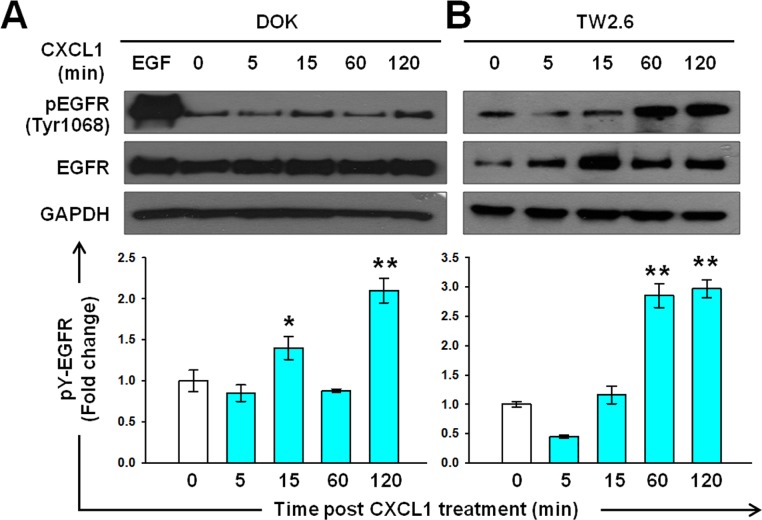
Induction of EGFR tyrosine phosphorylation by CXCL1 in DOK and TW2.6 cells Experimental conditions were similar to those described in Figure 2A, except DOK **A.** and TW2.6 **B.** cells were treated with 1 ng/mL of CXCL1 (blue bar) instead of IL-1β. A representative blot is shown in the top panel. Quantification of the blots was performed as described in Figure 2 and is presented as a bar chart in the bottom panel. Data are expressed as mean ± SD (*n* = 3). *P < 0.05 versus basal activation.

To investigate the role of the CXCL1-CXCR2 axis in IL-1β-mediated EGFR activation, we examine whether CXCL1 induces EGFR tyrosine phosphorylation in DOK and TW2.6 cells. In the DOK cells, an increased (approximately 1.5-fold high) EGFR tyrosine phosphorylation was observed at 15 min and further EGFR tyrosine phosphorylation was observed at 120 min (Figure 5A). In the TW2.6 cells, a reduction in EGFR tyrosine phosphorylation was observed at 5 min, followed by a gradual induction of approximately 3-fold at 120 min (Figure 5B).

**Figure 6:**
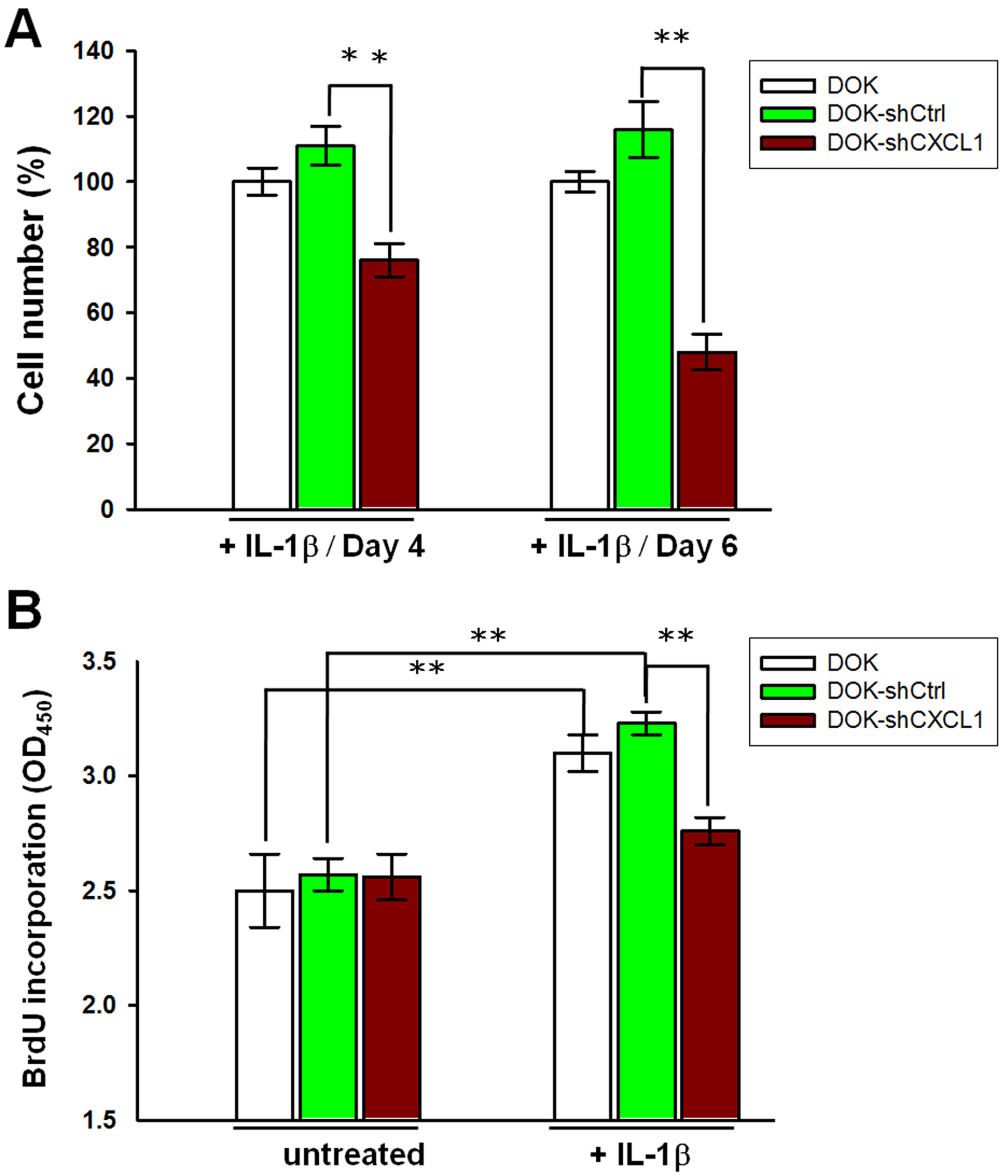
Knockdown of CXCL1 reduces IL-1β-mediated proliferation in DOK cell line DOK cells were infected with a nontargeting control vector (shCtrl) or CXCL1 shRNA (shCXCL1), inducing puromycin resistance. Cells were cultured for at least 2 weeks in the presence of puromycin (5 μg/mL) before the addition of 1 ng/mL of IL-1β. Cell proliferations were analyzed using MTT method **A.** or BrdU incorporation method **B.** as described in Figure 1. Data are expressed as mean ± SD of three independent experiments, where each experiment has been performed in sixplicates. **P < 0.01 versus the nontargeting control.

We then investigated whether CXCL1 contributed to IL-1β-mediated proliferation. DOK cells were infected with lentivirus carrying a CXCL1-targeting shRNA (shCXCL1) or nontargeting vector control (shCtrl) construct expressing a green fluorescent protein (GFP) and puromycin resistant gene. Cells were treated with puromycin for at least 2 weeks to ensure that the majority of cells (up to 95%) expressed the lentivirus constructs, which were assessed by GFP expression (Supplementary Figure S1A). Reduction in CXCL1mRNA expression and protein secretion were verified (Supplementary Figures S1B and S1C, respectively). The proliferation of nontargeting control cells (DOK-shCtrl) was slightly higher on IL-1β (1 ng/mL) addition than that of the uninfected (DOK) cells, whereas the inhibition of CXCL1 expression (DOK-shCXCL1) markedly reduced IL-1β-mediated DOK proliferation to 35% and 68% on day 4 and day 6, respectively, compared with the DOK-shCtrl cell proliferation after IL-1β addition (Figure 6A). Consistent with the MTT assay results, the BrdU assay revealed that, the BrdU incorporation rates in the DOK and DOK-shCtrl cells were significantly increased in response to IL-1β treatment (Figure 6B). In the presence of IL-1β, the BrdU incorporation rate in the DOK-shCXCL1 cells was lower than that in the DOK-shCtrl and DOK cells. In the absence of IL-1β (untreated), no significant difference was observed in the BrdU incorporation rate among the DOK-shCXCL1, DOK-shCtrl, and DOK cells (Figure 6B). These results indicated that CXCL1 contributed to IL-1β-mediated proliferation of DOK cells.

### CXCL1-CXCR2 axis is required for IL-1β-mediated EGFR tyrosine phosphorylation

**Figure 7:**
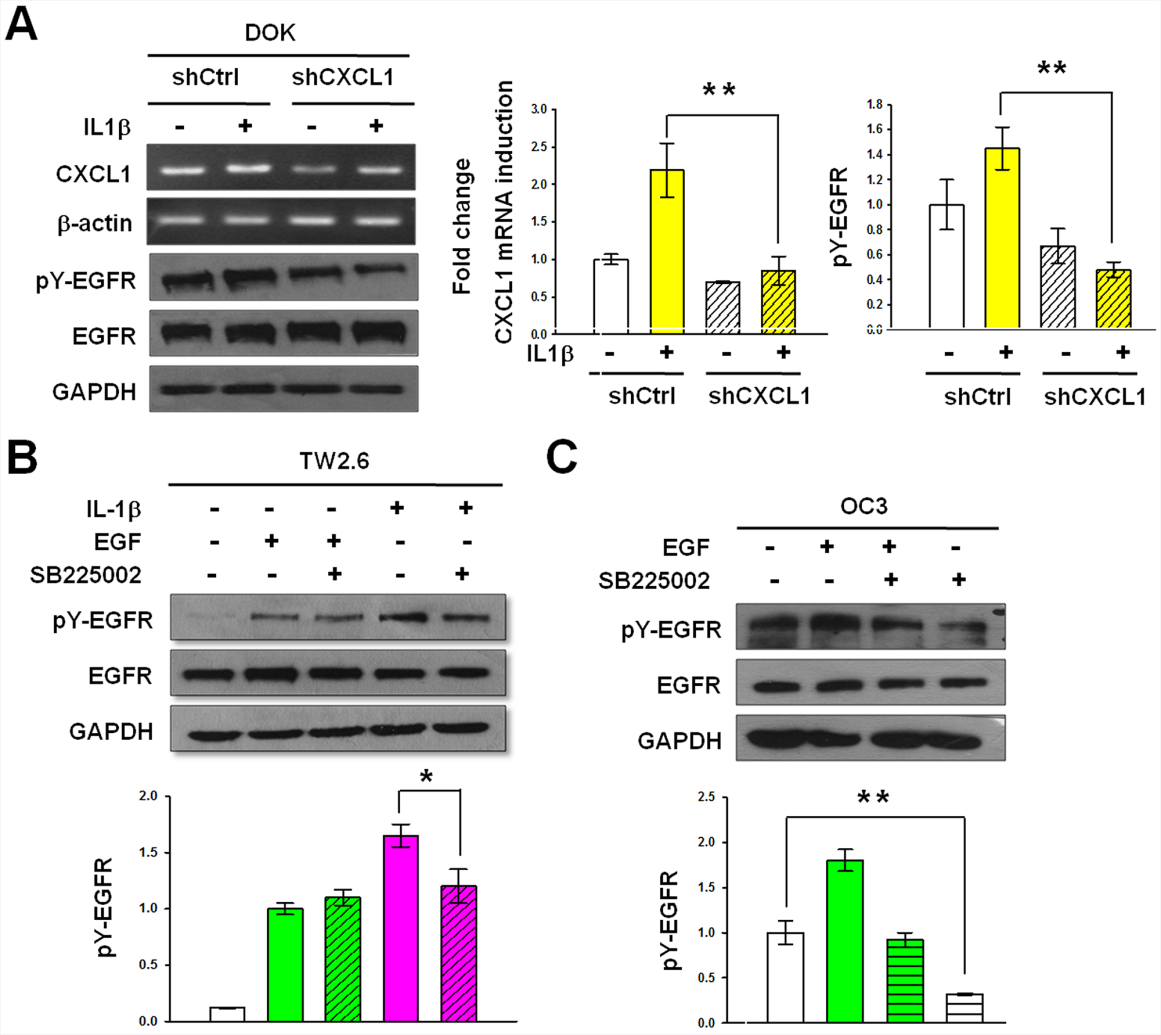
CXCL1 and CXCR2 are required for IL-1β-mediated EGFR tyrosine phosphorylation **A.** Knockdown of CXCL1 inhibits basal and IL-1β-mediated EGFR tyrosine phosphorylation. Nontargeting control cells (shCtrl) and CXCL1- targeting shRNA cells (shCXCL1) derived from DOK were established as described in Figure 6. After serum starvation, cells were treated with IL-1β (1 ng/mL). Cell lysates and total RNA were harvested 2 hours after IL-1β addition. CXCL1 mRNA expression and EGFR tyrosine phosphorylation were analyzed and quantified as described in Figures 3A and 2A, respectively. Representative blots are shown. Data are expressed as mean ± SD (*n* = 3). **P < 0.01 versus untreated or IL-1β-treated nontargeting control cells. **B.** Inhibition of IL-1β-mediated EGFR phosphorylation by CXCR2 antagonist SB225002. TW2.6 cells in serum-free medium were treated with 200 nM SB225002 for 20 min and followed by 1 ng/mL of IL-1β stimulation for 20 min. **C.** Inhibition of constitutive EGFR phosphorylation by SB225002. Experiments were conducted using OC3 cells following the procedure described in (B), except without IL-1β stimulation. EGFR tyrosine phosphorylation was analyzed and quantified as described in Figure 2A. Data are expressed as mean ± SD (*n* = 3), *P < 0.05 and **P < 0.01 versus IL-1β-mediated activation (B) or basal activation (C).

We showed that both CXCL1 and CXCR2 were expressed in DOK and OSCC cells (Figures 4C–4E), and CXCL1 could induce EGFR tyrosine phosphorylation (Figure 5). Therefore, we next investigated whether the CXCL1-CXCR2 axis was responsible for IL-1β-mediated EGFR tyrosine phosphorylation in DOK and OSCC cells. As shown in Figure 7A, lentivirus shRNA significantly inhibited IL-1β-induced CXCL1 expression. This reduction resulted in a significant reduction in the basal level and IL-1β-mediated EGFR tyrosine phosphorylation, whereas IL-1β-induced EGFR phosphorylation and CXCL1 expression remained markedly high in the nontargeting control cells. These results indicated that CXCL1 was involved in the IL-1β-mediated phosphorylation of EGFR. We then used the CXCR2 antagonist SB225002 to determine whether CXCL1 functions through CXCR2 to regulate IL-1β-mediated EGFR phosphorylation. TW2.6, an OSCC cell line with a relatively low level of endogenous IL-1β, was pretreated with SB225002 (200 nM) for 20 min, followed by IL-1β treatment for 20 min before EGFR tyrosine phosphorylation analysis. As shown in Figure 7B, the TW2.6 cells treated with SB225002 exhibited a 45% reduction in IL-1β-mediated EGFR tyrosine phosphorylation, whereas EGF-induced EGFR phosphorylation was unaffected by SB225002 treatment, indicating that CXCR2 was involved in IL-1β-mediated EGFR phosphorylation but not in that induced by EGF. The OC3 cells, which highly secrete IL-1β, were preincubated with SB225002 (200 nM) for 20 min before estimating EGFR tyrosine phosphorylation by western blotting. As shown in Figure 7C, the constitutive EGFR phosphorylation of OC3 cells was significantly inhibited by SB225002, suggesting that CXCR2 signaling contributes to the intrinsic EGFR activation of this IL-1β-secreting cell line. EGF increased the phosphorylation of EGFR by 1.9- ± 0.2-fold compared with that at the basal level. Preincubation of SB225002 reduced EGFR phosphorylation in EGF-treated OC3 cells, which might be possibly because of IL-1β-mediated constitutive EGFR phosphorylation inhibition by SB225002. Overall, these results suggested that the CXCL1-CXCR2 axis was responsible for the IL-1β-mediated EGFR tyrosine phosphorylation in DOK cells and OSCC.

**Figure 8:**
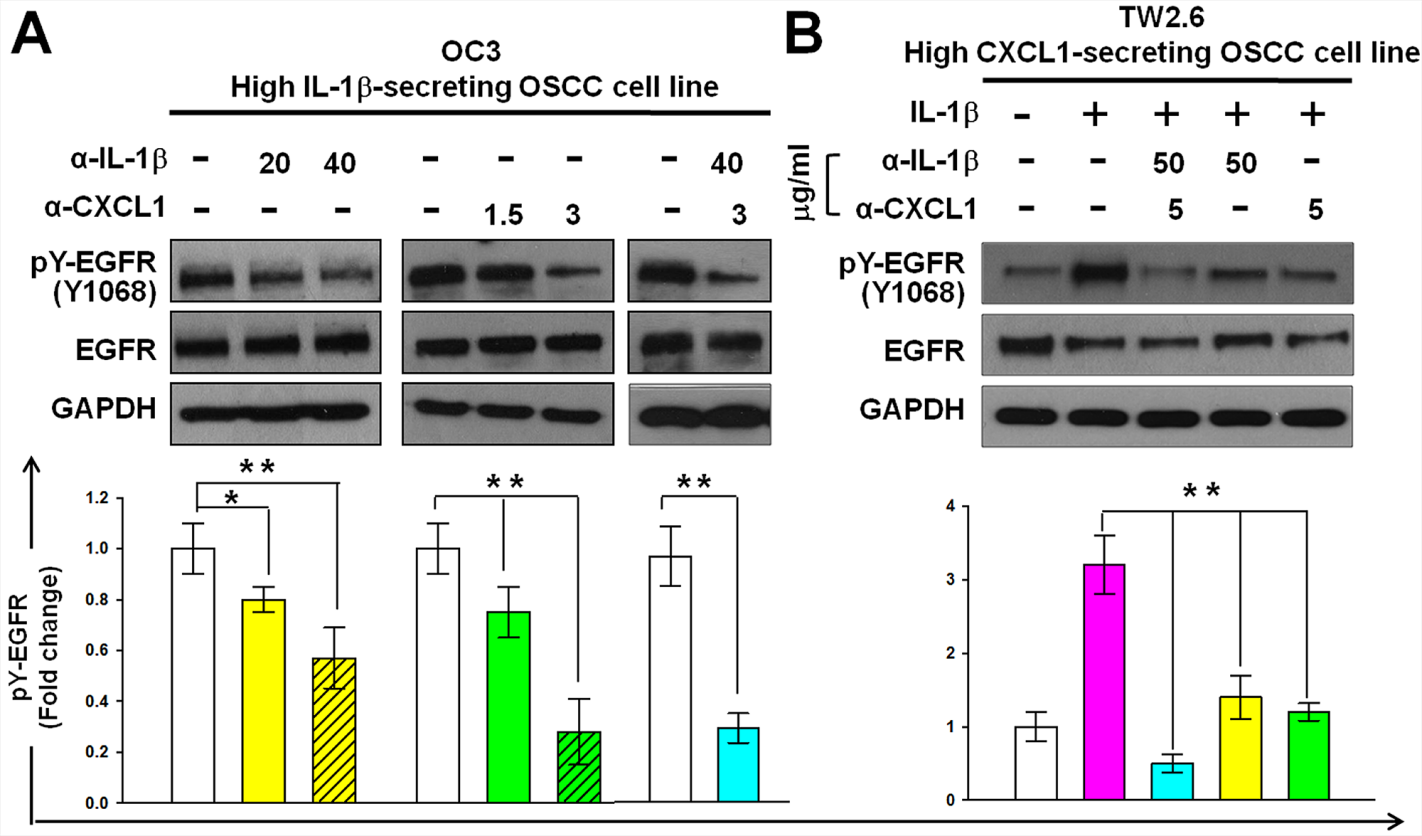
Reduction of CXCL1 or IL-1β activities reduces the phosphorylation of EGFR in OSCC cells Inhibition of constitutive **A.** and IL-1β-mediated **B.** EGFR phosphorylation using neutralizing antibodies against IL-1β or CXCL1 in OC3 and TW2.6 cells, respectively. Cells were deprived of serum for 24 hours. After incubating neutralizing antibodies against IL-1β or / and CXCL1 for 4 hours and then without (A) or with (B) IL-1β (1 ng/mL) stimulation for 30 min, EGFR tyrosine phosphorylation was analyzed and quantified as described in Figure 2A. Data are expressed as mean ± SD (*n* = 3). *P < 0.05 and **P < 0.01 versus basal activation (A) or IL-1β-mediated activation (B).

To confirm the role of IL-1β and CXCL1 in the EGFR activation of OSCC, we inhibited their biological activities by using neutralizing antibodies to examine the effect of these molecules on EGFR tyrosine phosphorylation. OC3 or TW2.6 cells were incubated with neutralizing antibodies against IL-1β, CXCL1, or both for 4 hours. Subsequently, TW2.6 cells alone were treated with IL-1β (1 ng/mL) for 30 minutes. The incubation of neutralizing antibodies against IL-1β (20 or 40 μg/mL) or CXCL1 (1.5 or 3 μg/mL) in the high IL-1β-secreting OC3 cells inhibited constitutive EGFR phosphorylation in a dose-dependent manner, and the combined use of two antibodies was more effective than that of a single antibody in inhibiting constitutive EGFR phosphorylation (Figure 8A). In the high CXCL1-secreting TW2.6 cells (Figure 4C), the incubation of neutralizing antibodies against CXCL1 (5 μg/mL) inhibited the IL-1β-mediated EGFR phosphorylation significantly, and the neutralizing antibodies against IL-1β (50 μg/mL), alone or in combination with anti-CXCL1 (5 μg/mL), inhibited IL-1β-mediated EGFR phosphorylation (Figure 8B). These results suggested that IL-1β and CXCL1 contribute to constitutive and IL-1β-mediated phosphorylation of EGFR in OC3 and TW2.6 cells, respectively.

## DISCUSSION

**Figure 9:**
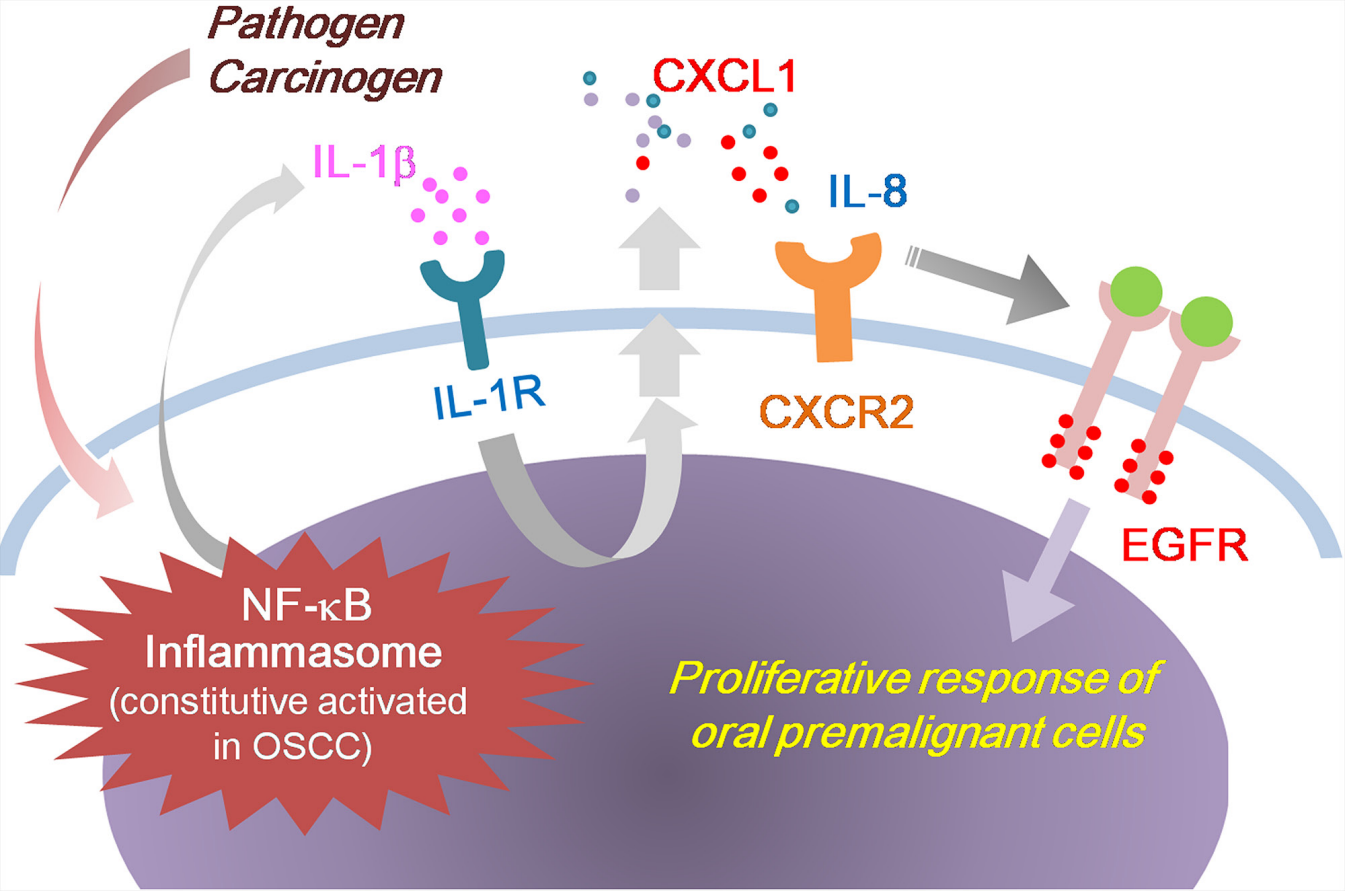
Model outlining the role of IL-1β in the regulation of EGFR signaling and oral malignant transformation Tumor-derived IL-1β induces CXCL1 production, which in turn activates EGFR through CXCR2, causing a proliferative response of oral premalignant cells.

Chronic inflammation is associated with the development of OSCC. We previously identified that IL-1β secretion is high in OSCC cells, and IL-1β is involved in the initial and late stages of oral malignancy [5]. In this study, we showed that IL-1β induces CXCL1 production, which in turn activates EGFR through CXCR2, causing a proliferative response of oral premalignant cells (Figure 9). Our findings revealed a novel oncogenic pathway for OSCC-associated risk factors. In this cancer-promoting process, IL-1β and CXCL1-CXCR2 have crucial regulating roles in integrating inflammation stimuli and an EGFR signaling cascade. The CXCL1-CXCR2 axis may serve as a transmitter to transduce the inflammasome-derived IL-1β signal into the signal network modulated by EGFR. Because of the involvement of dysregulated EGFR signaling and an inflammatory microenvironment in oral tumorigenesis, converging these 2 oncogenic events has a profound effect on the etiology of OSCC and the clinical management of this cancer.

As a crucial determiner of inflammation, the biological processes of IL-1β secretion are tightly regulated. Both activating NF-κB and inflammasomes are essential for the production of IL-1β [27, 28]. An inflammasome is formed by multiprotein complex including pattern recognition receptors (PRRs) which sense and respond to conserved pathogen motifs and endogenous molecules released upon cell damage or stress [27]. Thus, according to our findings, a broad spectrum of pathogens or molecules recognized by the PRRs could induce the EGFR signaling cascade through trigger IL-1β production, and such that the cascade exerts its tumor-promoting role. Our previous work demonstrated that NNK induces IL-1β production in OSCC cells [5]. Lemjabbar et al provided supporting evidence for our hypothesis [29]. Their work associated EGFR signal transactivation with increased cell proliferation in human lung carcinoma in response to cigarette smoking. According to our findings, cigarette smoking could possibly induce IL-1β in lung carcinoma and transactivate EGFR through CXCL1-CXCR2 axis. GPCRs represent the largest group of cell-surface receptors and exert a wide variety of biological functions. In the last decade, several investigations have demonstrated that the inter-receptor cross-talk mechanism combines the broad diversity of GPCRs with the signaling capacities of EGFR, and this association is frequently observed in human cancer. Transactivation of EGFR by GPCR agonists has been demonstrated in HNCS cell lines by Gschwind et al [30]. CXCR2 is activated by cancer-promoting IL-8 and CXCL1 and could transactivate EGFR in ovarian cancer and non-small cell lung cancer [22–24]. This receptor has been shown to be widely expressed in patients with OSCC [39] and has been reported to be crucial for aggressive phenotypes of this cancer [31–33]. Our data demonstrate that CXCR2 contributes to IL-1β-mediated EGFR transactivation in OSCC and may provide an explanation for the oncogenic effect of CXCR2 in this cancer. Additionally, increasing evidence shows a direct correlation between aberrant GPCR signaling and the onset of hyperproliferative disorders [30, 34, 35]. As increased proliferation may be a critical initiation step in carcinogenesis, the promoting effect of IL-1β on the proliferation of DOK cells is meaningful to oral malignant transformation. We showed that the IL-1β-mediated EGFR transactivation involving the CXCL1-CXCR2 axis was implicated in DOK proliferation in the presence of IL-1β (Figure 1 and 6). These results suggest that the CXCL1-CXCR2 axis-targeting therapy could be considered a prevention strategy for patients with chronic inflammatory oral lesions from developing OSCC.

In the OC3 cell line, following the strong IL-1β-induced activation within 40 minutes, the EGFR phosphorylation attenuated 60 minutes after IL-1β addition (Supplementary Figure S2), and the CXCL1 levels remained high simultaneously. This trend may be explained by a negative feedback regulation mechanism of EGFR in this cell line. Er et al., [36] reported that Akt-mediated endocytic trafficking of EGFR for promoting its degradation is likely to be a common feedback mechanism for terminating EGFR signaling and reducing receptor abundance. In our previous study [5], IL-1β stimulated Akt activation in DOK and OSCC cells, thus facilitating a negative feedback regulation mechanism. In addition, some other negative regulators of EGFR may be induced by the strong activation of its downstream signaling pathways. Wang et al. [37] demonstrated that PHLDA2 was robustly induced by the EGFR signaling pathways, and PHLDA2 mediates a negative feedback loop, inhibiting the activation of EGFR and Akt. The regulation network of EGFR in OC3 cells warrants further investigation. CXCL1 expression is markedly upregulated by MAPK/ERK signaling in human decidua [38]. This finding is consistent with that of our study, in which the intrinsic and IL-1β-induced-CXCL1 expression was abolished by the MAPK inhibitor U0126 in DOK and TW2.6 cells (Supplementary Figure S3A). Furthermore, intrinsic phosphorylated Erk1/2 were higher in the TW2.6 cells than in the OC3 cells (Supplementary Figure S3B), and this may have resulted in the high CXCL1 expression in TW2.6 cells, despite lower IL-1β levels in the TW2.6 cells than in the OC3 cells (Figure 4C). Therefore, although the IL-1β antibodies neutralized IL-1β in the culture medium and suppressed IL-1β-induced CXCL1 production, the tyrosine phosphorylation of EGFR stimulated by the intrinsic activated CXCL1-CXCR2 axis remains substantial in the TW2.6 cells (Figure 8B). Furthermore, SB225002 or neutralizing antibodies against CXCL1 reduced IL-1β-mediated EGFR tyrosine phosphorylation more efficiently compared with IL-1β antibodies (Figures 7B and 8B). These findings imply that a CXCL1–CXCR2 axis-targeted drug may overcome EGFR activation more efficiently compared with an IL-1β-targeted regimen under therapeutic intervention. In mammalian cells, at least seven ligands for EGFR have been identified: EGF, TGF-α, HB-EGF, amphiregulin, betacellulin, epigen, and epiregullin. HB-EGF is synthesized in a soluble, membrane-anchored form (pro-HB-EGF) and binds to EGFR through ectodomain shedding. Takenobu et al. [39] reported that IL-1β triggered ectodomain shedding of pro-HB-EGF in vero cells. This mechanism may occur in OSCC cells and explain why the CXCR2 inhibitor SB225002 cannot completely inhibit IL-1β-mediated EGFR tyrosine phosphorylation (Figure 7B). Furthermore, we cannot exclude the possibility that EGF-like growth factors other than HB-EGF can be induced by IL-1β and can account for the prolonged EGFR tyrosine phosphorylation after SB225002 treatment.

EGFR is considered a suitable drug target for OSCC treatment because the majority of OSCC cells overexpressed EGFR [40], and high EGFR tumor levels are associated with poor clinical outcomes [41]. However, several clinical trials on EGFR-targeted therapies, including EGFR-directed antibodies and TKIs, such as erlotinib, gefitinib, and afatinib, have reported that only a small subset of patients with OSCC responded to these treatments [42–44]. Several resistance mechanisms to EGFR inhibitors are clinically validated in lung and colon cancers and are likely applicable to OSCC. These recognized resistance mechanisms to EGFR-targeted therapies may be classified into four categories [45]: target modification, an EGFR mutation to a drug-resistant state. In several patients with non-small cell lung cancer, resistance develops through an acquired activating EGFR mutation, T790M [46]; oncogenic shift or accessory pathway activation [47], such as MET amplification, KRAS activation, and PI3K-Akt pathway mutations; PIK3CA mutations have been associated with resistance to EGFR-targeted monoclonal antibodies in patients with metastatic colorectal cancers. PIK3CA mutations were identified in up to 57.1% of OSCC lines and 16.7% of OSCC tumors [49]; and impairment of the apoptotic pathway essential for EGFR TKI-mediated apoptosis, such as germline BIM deletion [50], histological transformation, or an epithelial–mesenchymal transition [51]. Current clinical approaches to overcome resistance in lung adenocarcinoma are the use of irreversible and mutant-selective EGFR inhibitors, and combining an EGFR inhibitor with a drug targeting a resistance pathway, such as the combination of erlotinib and an MET inhibitor. According to our model, upregulated IL-1β and CXCL1 expression may provide a rationale for constitutive EGFR activation frequently observed in OSCC, and, therefore, IL-1β and CXCL1 are promising molecular markers for identifying OSCC patients who respond to EGFR-targeted therapy. In addition, a combination of EGFR TKIs with a regimen targeting the CXCL1-CXCR2 axis may be a promising therapeutic strategy for OSCC patients with over-expressed EGFR.

Future investigation must aim at identifying the molecules responsible for EGFR transactivation caused by CXCR2 in OSCC. Because evidence indicates the importance of EGFR signal transactivation and inflammation in this malignancy, fully elucidating these signaling mechanisms will provide further insight into oral carcinogenesis, and the components involved represent promising targets for therapeutic intervention.

## MATERIALS AND METHODS

### Chemicals

AG1478, Erlotinib, and SB225002 were purchased from ApexBio, Biovision, and Enzo Life Science, respectively. Recombinant human IL-1β, CXCL1, and IL-1Ra was purchased from R&D System. Western blotting analysis was performed using antibodies against human EGFR and phosphorylated EGFR (pEGFR) (Y1068) (Cell Signaling). Neutralizing antibodies experiments were performed using antibodies against human IL-1β (Acris Antibodies) and human CXCL1 (RayBiotech).

### Cell lines and cell culture

Human dysplastic oral keratinocytes DOK [52] were maintained in DMEM supplemented with 10% fetal calf serum, 100 g/ml penicillin, and 100 g/ml streptomycin. Human OSCC cells TW2.6 and OC3 were cultured as previously described [53].

### RNA extraction and real-time quantitative PCR (qPCR)

Extraction of cellular total RNA, synthesis of cDNA from total RNA and qPCR were performed as previously described [5, 53]. The primer sequences for qPCR are as follows: 5′-TCCTGCATCCCCCATAGTTA-3 (forward) and 5′-CTTCAGGAACAGCCACCAGT-3′ for human CXCL1; 5′-GAGGCACAGTGAAGACATCG-3′ (forward) and 5′-GCTGGGCTTTTCACCTGTAG-3′for human CXCR2; and 5′-CCAACCGCGAGAAGATGA-3′ (forward) and 5′-CCAGAGGCGTACAGGGATAG-3′ for β-actin (ACTB). The primer sequences for human pro- IL-1β were as previously described [5].

### Knockdown of CXCL1

For knockdown of CXCL1 expression, we purchased GIPZ lentivirus particles carrying shRNA specifically targeting CXCL1 from Dharmacon. To cell infection, 50% confluent of DOK cells were incubated with lentivirus for 6 hour, and the medium containing puromycin (5 μg/ml; Sigma-Aldrich) was replaced to select stable cell pools for at least 2 weeks before usage. Cells infected with lentivirus with empty vector (shCtrl) were used as controls.

### MTT proliferation assay

Cells were seeded at 1 × 10^3^ per well on 96-well plates. 10 μl of MTT ([3-(4,5-dimethylthiazol-2-yl)-2, 5-diphenyl tetrazolium bromide], 5 mg/ml, Sigma-Aldrich) was added to each well at indicated time; the cells were further incubated for 4 hour at 37°C and then subcultured in the medium with 100 μl of DMSO. The absorbance at 540 nm was measured on a micro ELISA reader (Bio-Rad).

### BrU cell proliferation assay

Cells were seeded in five replicates in 96-well plates at a density of 2 × 10^4^ cells per well. After adhesion, cells were cultured in a standard culture medium or treated with indicated concentrations of AG1478 or erlotinib or vehicle alone as control for 4 hours. Cell proliferation was evaluated by measuring BrU incorporation during DNA synthesis according to the manufacturer's instructions (Merck Millipore). BrDU incorporation was measured using chemiluminescence (absorbance: 450 nm).

### Western blot analysis

Western blotting analysis was carried out using 30 μg of proteins extracted from cultured cells, separated by SDS-PAGE, and transferred onto nitrocellulose membranes. Membranes were blocked and hybridized with relevant antibodies. ECL chemiluminescence (EMD, Millipore) was used to detect the horseradish peroxidase-conjugated secondary antibodies. Anti-EGFR was used at 1 : 2000, and anti-pEGFR (Y1068) at 1 : 500.

### ELISA

Cells were seeding in a 24-well culture plate (2 × 10^5^cells/well) and grown in 2 ml of serum free medium for 16 hour. The supernatants were collected and the secreted IL-1β or CXCL1 was quantified using ELISA for human IL-1β or CXCL1 (R&D System) following the manufacturer's protocol. Concentrations of measured IL-1β or CXCL1 were normalized to the cell number determined in parallel.

### Immunofluorescence cell staining

We determined the expression of CXCR2 protein by indirect immunofluorescence staining. Cells were seeded on glass coverslips in 6-well plates and grown to 70% confluence. Cells were fixed with 4% paraformaldehyde at room temperature for 10 minutes and then blocked with 1% BSA for 1 hour. The cells were incubated with antibodies against human CXCR2 (Novus Biologicals) overnight at 4°C, washed three times in PBS followed by incubation with secondary antibodies conjugated with Texas Red (Santa Cruz Biotechnology). The DNA dye DAPI (BD Biosciences) was used to stain DNA.

### Immunohistochemistry

The tongue tissues were fixed overnight in formalin, embedded in paraffin, and sectioned at 5 μm thickness. Sections were deparaffined and rehydrated using a xylene-alcohol series protocol. Endogenous peroxidase activity was quenched using 3% (v/v) hydrogen peroxide-methanol (1:1 v/v) solution at room temperature for 10 minutes. Heat-induced antigen retrieval was performed in citrate buffer(0.01 M, pH6.0) at pressure cooker (Cell Marque) to expose antigen for 15 min. Sections were then incubated overnight at 4°C with either of the following primary antibodies: rabbit polyclonal anti-IL-1β (1:500, Abcam) and rabbit polyclonal anti-CXCL1 (1:1000, Abcam). A peroxidase-conjugated polymer reagent (REAL™ EnVision™ Detection System, DAKO) with a diaminobenzidine (DAB, DAKO) chromogen was then used to develop the stain. Sections were counterstained with hematoxylin and then dehydrated and mounted. Controls consisted of omission of the primary antibody.

### Statistical analysis

Comparison of the results between various experimentally treated groups and their corresponding controls was carried out by Student's *t*-test. All comparisons were considered significant when *p* < 0.05.

## SUPPLEMENTARY FIGURES


