# Supplementary material for: Interleukin-1 beta transactivates epidermal growth factor receptor via the CXCL1-CXCR2 axis in oral cancer
Source: Oncotarget. 2015 Oct 9;6(36):38866–80. doi: 10.18632/oncotarget.5640 (PMC4770743; doi:10.18632/oncotarget.5640)
Supplement: Supplementary file 1 [file oncotarget-06-38866-s001.pdf]

SUPPLEMENTARY FIGURES

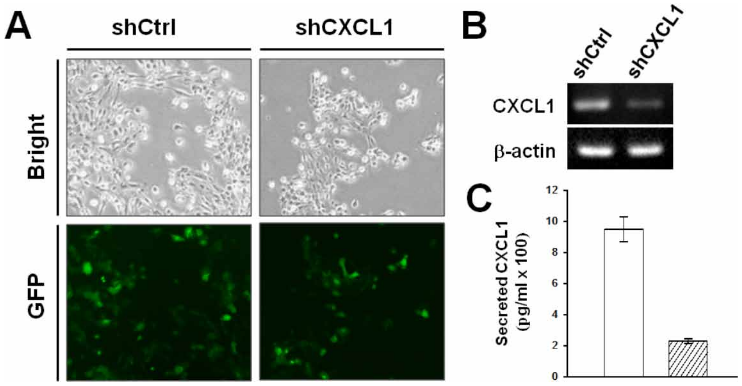

Supplementary Figure S1: Expression of CXCL1 is suppressed efficiently in DOK cells by lentivirus-mediated shRNA method. A. Representative images of DOK cells infected with lentivirus carrying shRNA targeting CXCL1 (shCXCL1) or non-targeting control vector (shCtrl). Bright, bright field. **B.** Quantitative RT-PCR analyzed mRNA levels of CXCL1 in DOK-shCXCL1 and DOK-shCtrl cells. **C.** ELISA analysis of secreted protein levels of CXCL1 in in DOK-shCXCL1 and DOK-shCtrl cells.

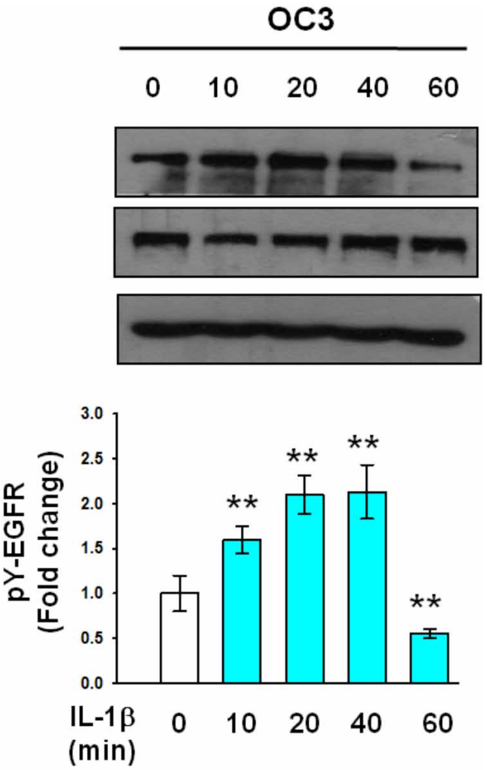

Supplementary Figure S2: Kinetics of EGFR tyrosine phosphorylation in response to IL-1β in OC3 cell line.

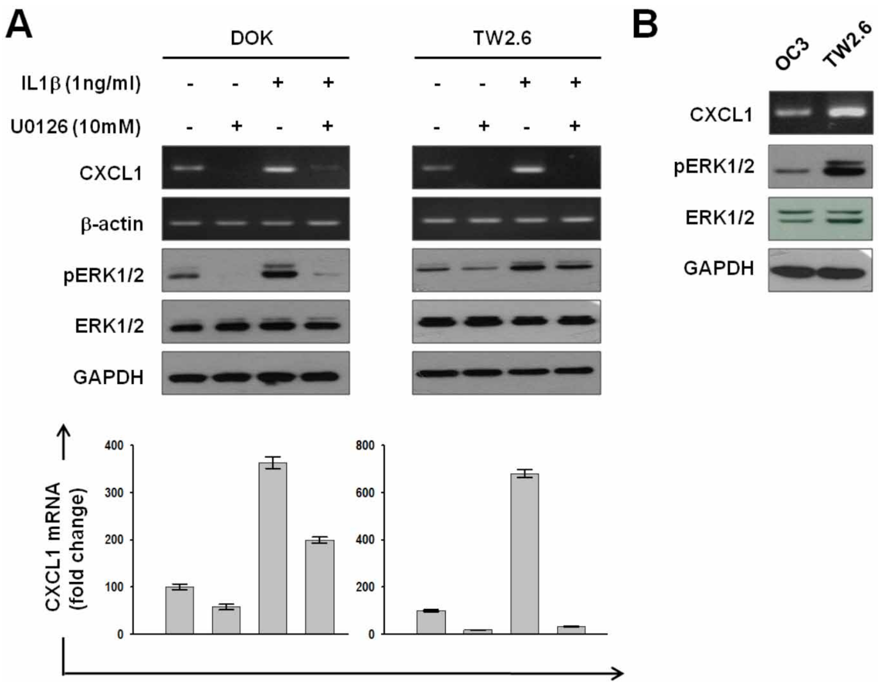

Supplementary Figure S3: MAPK signaling pathway is responsible for CXCL1 production in DOK and OSCC cell lines. A. MAPK inhibitor U0126 inhibited the constitutive and IL-1β-induced CXCL1 expression. **B.** Correlation between phosphorylated ERK1/2 and CXCL1 expression in OSCC cell lines.
